# Time Trends of Acrylamide Exposure in Europe: Combined Analysis of Published Reports and Current HBM4EU Studies

**DOI:** 10.3390/toxics10080481

**Published:** 2022-08-17

**Authors:** Michael Poteser, Federica Laguzzi, Thomas Schettgen, Nina Vogel, Till Weber, Philipp Zimmermann, Domenica Hahn, Marike Kolossa-Gehring, Sónia Namorado, An Van Nieuwenhuyse, Brice Appenzeller, Thórhallur I. Halldórsson, Ása Eiríksdóttir, Line Småstuen Haug, Cathrine Thomsen, Fabio Barbone, Valentina Rosolen, Loïc Rambaud, Margaux Riou, Thomas Göen, Stefanie Nübler, Moritz Schäfer, Karin Haji Abbas Zarrabi, Liese Gilles, Laura Rodriguez Martin, Greet Schoeters, Ovnair Sepai, Eva Govarts, Hanns Moshammer

**Affiliations:** 1 Department of Environmental Health, Center for Public Health, Medical University of Vienna, 1090 Vienna, Austria; 2 Unit of Cardiovascular and Nutritional Epidemiology, Institute of Environmental Medicine, Karolinska Institute, Nobels väg 13, Box 210, 17177 Stockholm, Sweden; 3 Institute for Occupational, Social and Environmental Medicine, Medical Faculty, RWTH Aachen University, Pauwelsstrasse 30, D-52074 Aachen, Germany; 4 German Environment Agency (UBA), D-14195 Berlin, Germany; 5 Department of Epidemiology, National Institute of Health Dr. Ricardo Jorge, 1649-016 Lisbon, Portugal; 6 Laboratoire National de Santé (LNS), L-3555 Luxembourg, Luxembourg; 7 Department of Precision Health, Luxembourg Institute of Health (LIH), L-4354 Luxembourg, Luxembourg; 8 Faculty of Food Science and Nutrition, School of Health Sciences, University of Iceland, 102 Reykjavik, Iceland; 9 Department of Pharmacology and Toxicology, University of Iceland, 107 Reykjavik, Iceland; 10 Norwegian Institute of Public Health, Lovisenberggata 8, 0456 Oslo, Norway; 11 Department of Medical Area, DAME, University of Udine, 33100 Udine, Italy; 12 Institute for Maternal and Child Health-IRCCS “Burlo Garofolo”, 34137 Trieste, Italy; 13 Santé Publique France, French Public Health Agency (ANSP), 94415 Saint-Maurice, France; 14 Institute and Outpatient Clinic of Occupational, Social and Environmental Medicine, Friedrich-Alexander Universität Erlangen-Nürnberg, Henkestraße 9-11, D-91054 Erlangen, Germany; 15 VITO Health, Flemish Institute for Technological Research (VITO), 2400 Mol, Belgium; 16 UK Health Security Agency, London SE1 8UG, UK; 17 Department of Hygiene, Medical University of Karakalpakstan, Nukus 230100, Uzbekistan

**Keywords:** acrylamide, glycidamide, exposure level, time-trend, human biomonitoring, HBM

## Abstract

More than 20 years ago, acrylamide was added to the list of potential carcinogens found in many common dietary products and tobacco smoke. Consequently, human biomonitoring studies investigating exposure to acrylamide in the form of adducts in blood and metabolites in urine have been performed to obtain data on the actual burden in different populations of the world and in Europe. Recognizing the related health risk, the European Commission responded with measures to curb the acrylamide content in food products. In 2017, a trans-European human biomonitoring project (HBM4EU) was started with the aim to investigate exposure to several chemicals, including acrylamide. Here we set out to provide a combined analysis of previous and current European acrylamide biomonitoring study results by harmonizing and integrating different data sources, including HBM4EU aligned studies, with the aim to resolve overall and current time trends of acrylamide exposure in Europe. Data from 10 European countries were included in the analysis, comprising more than 5500 individual samples (3214 children and teenagers, 2293 adults). We utilized linear models as well as a non-linear fit and breakpoint analysis to investigate trends in temporal acrylamide exposure as well as descriptive statistics and statistical tests to validate findings. Our results indicate an overall increase in acrylamide exposure between the years 2001 and 2017. Studies with samples collected after 2018 focusing on adults do not indicate increasing exposure but show declining values. Regional differences appear to affect absolute values, but not the overall time-trend of exposure. As benchmark levels for acrylamide content in food have been adopted in Europe in 2018, our results may imply the effects of these measures, but only indicated for adults, as corresponding data are still missing for children.

## 1. Introduction

Representing a pervasive food contaminant, acrylamide has been recognized as a concern for public health since a potentially increased risk for cancer has been attributed to acrylamide intake [1,2]. Acrylamide is mainly formed in a Maillard-reaction, a heat induced interaction of the amino-acid asparagine with a carbonyl-containing moiety, as provided by oxidized starch [3], but it is also present in tobacco smoke. Acrylamide is found in products like cereals [4], bakery [5], dried fruits, olives [6] and coffee [7]. Acrylamide as well as its liver metabolite glycidamide are able to interact with DNA [8] and both are thus considered as potential carcinogenic substances [9,10]. Several other adverse health effects were recognized in connection with acrylamide intake, including neurotoxicity [11,12] and impaired fertility [13].

Since recognition of acrylamides as food contaminants [1], a number of independent human biomonitoring studies have been performed, worldwide and in Europe, with the aim to quantify the actual acrylamide exposure as well as main intake determinants in the general population of several countries.

Individual exposure to a substance contained in food items can be investigated either by calculating external intakes and combining data on consumption with measured concentrations of acrylamide in relevant food items, or by human biomonitoring and analyzing specific biomarkers that can be detected in the human body. For quantification of intake by food items, studies mainly rely on questionnaire-based estimations and are thus affected by common limitations of these methods [14,15,16]. Human biomonitoring provides an objective snapshot of current exposure resulting from different exposure pathways, but requires a laborious sampling process. While both methods of quantification have specific limitations, human biomonitoring does provide more accurate information on current individual exposure levels.

Acrylamide exposure can be quantified by human biomonitoring using either blood [17] or urine [18] samples. In blood, acrylamide and glycidamide can be detected in the form of adducts, covalently bound to valine residues of hemoglobin (AAVal, N-(2-carbamoylethyl)valine and GAVal, N-(2-carbamoyl-2-hydroxyethyl)valine) and in urine the mercapturic acids of acrylamide (AAMA, N-acetyl-S-(carbamoylethyl)-l-cysteine) as well as its epoxide metabolite glycidamide (GAMA, N-acetyl-S-(1-carbamoyl-2-hydroxyethyl)-l-cysteine) can be quantified, using high performance liquid chromatographic (HPLC) or gas chromatographic (GC) separation methods and subsequent mass spectrometry [19].

Acrylamide is typically taken up orally, either in form of dietary sources, in drinking water or by smoking with a (dietary) bioavailability (shown in rats) of about 30–44% [20]. After uptake, acrylamide is either metabolized by parts of the cytochrome P450 oxidase system (CYP2E1) to form glycidamide (GA) or it is directly reacting with glutathione to form N-acetyl-S-(2-carbamoylethyl)cysteine (AAMA). GA is able to react with glutathione to form the mercapturic acids N-Acetyl-S-2-(2-hydroxy-2-carbamoylethyl)cysteine (GAMA) and N-Acetyl-S-(1-carbamoyl-2-hydroxyethyl)cysteine. While GAMA levels are, additionally to GA concentrations, dependent on the efficacy of the cytochrome P450 oxidase system responsible for GA synthesis, AAMA levels may be regarded as a rather direct indicator of exposure. AAMA has been reported to consistently represent about 50% of the administered dose of acrylamide [21,22,23]. Toxicokinetics are however different for AAMA and GAMA, as indicated by a slightly delayed excretion by GAMA as compared to AAMA [22]. Biomonitoring of GAMA is nevertheless of high interest for public health, as glycidamide represents the reactive epoxide metabolite of acrylamide and is thus mainly determining cancerogenicity as well as non-cancerogenic toxicity of acrylamide in humans. Thus, AAMA and GAMA are both relevant for the estimation of acrylamide exposure related cancer risk, but are not necessarily expected to show the same ratio in all exposure settings and populations.

Other than urine biomarkers, blood adduct levels like AAVal and GAVal are considered as indicators of long-term exposure. While the elimination of acrylamide via urine has been described with a half-life of hours for AAMA [22,23] and up to days for GAMA [24], AAVal and GAVal have been reported (in rats) with half-lives of 12–13 days and 11–12 days respectively [25]. Due to these differences in elimination kinetics, simultaneous measurements of blood and urine levels are not well correlated.

First epidemiological results based on these biomarkers of acrylamide exposure indicated the requirement of regulatory measurements to limit potential health risks in the European population. Already as early as 2002, some sectors of the European food industry adopted voluntary efforts to mitigate the acrylamide content in their products, as later outlined in the Acrylamide Toolbox (2005–2019) [26] and FAO Code of Practice (2009) [27]. Between 2007 and 2019, EU member states monitored acrylamide in food based on recommendations of the EU Commission (CR 2007/331/EC, CR 2010/307/EC, CR 2013/647/EU, CR 2019/1888/EU). In addition, monitoring data has been collected by the European Food Safety Agency (EFSA), providing the basis for “indicative values” for concentrations found in food products (European Regulation November 2011) that have been updated in 2013 with the consequence that some of the initially determined indicative values have been lowered. Several studies have been performed to assess the effectiveness of these measures on food source level, analyzing the average acrylamide content in different categories of food in Europe after mitigation measures were put into action. An EFSA report from 2012 [28] showed a slight decrease of average acrylamide content in industrial dietary products during the period 2007–2010 for some of the food categories, while for other the content of acrylamide was not changed.

In June 2015, EFSA released its first full risk assessment on acrylamide in food [2], officially recognizing it as a potentially carcinogenic substance and concluding that exposure levels in the European population did not drop significantly since the initial recognition of the risk. This assessment of acrylamide genotoxicity was extended in 2022 [29] where methods and conclusions of the initial risk assessment were confirmed. Commission Regulation 2017/2158 [30] established mitigation measures and benchmark levels for the reduction of acrylamide content in food, adopted in 2017 and put in force in April 2018.

As acrylamide and its metabolite glycidamide were shown to be potentially genotoxic and cancerogenic, EFSA concluded that a tolerable daily intake (TDI) for acrylamide cannot be set. Instead, a dose range was estimated within which acrylamide is likely to cause a measurable increase in tumor incidence or other non-cancer effects, (reproduction and developmental defects, neurotoxicity) [31]. The lower limit of this range is represented by the Benchmark Dose Lower Confidence Limit (BMDL_10_) (including 10% additional risk for tumors) and has been determined for acrylamide and cancerogenicity related outcomes at 0.17 mg/kg bw/day (EFSA 2105 [2]).

HBM4EU (Human BioMonitoring for the European Union [32,33] represents a multinational scientific project, established to gain knowledge about the overall burden of specific pollutants and contaminants within the European population using human biomonitoring. The HBM4EU Aligned Studies (studies collaborating on aligning human biomonitoring studies in the general population with combined financing from countries and HBM4EU, 2014–2021), provided biomonitoring information on acrylamide exposure for a recent time period.

Availability of comparable acrylamide exposure values in European populations provided by HBM4EU Aligned Studies, representing several years in total, opened the possibility to review these recent findings in context with previously reported European acrylamide biomarker levels, to obtain an overall long-term time-trend of exposure. Therefore, we here set out to provide a summarizing view of the overall time-trend of acrylamide biomarker levels from a harmonized set of European studies since 2001, including previously published papers and recent results from HBM4EU Aligned studies and by integrating a maximal number of available data sources after harmonization (see Section 2.4).

## 2. Materials and Methods

**Figure 1:**
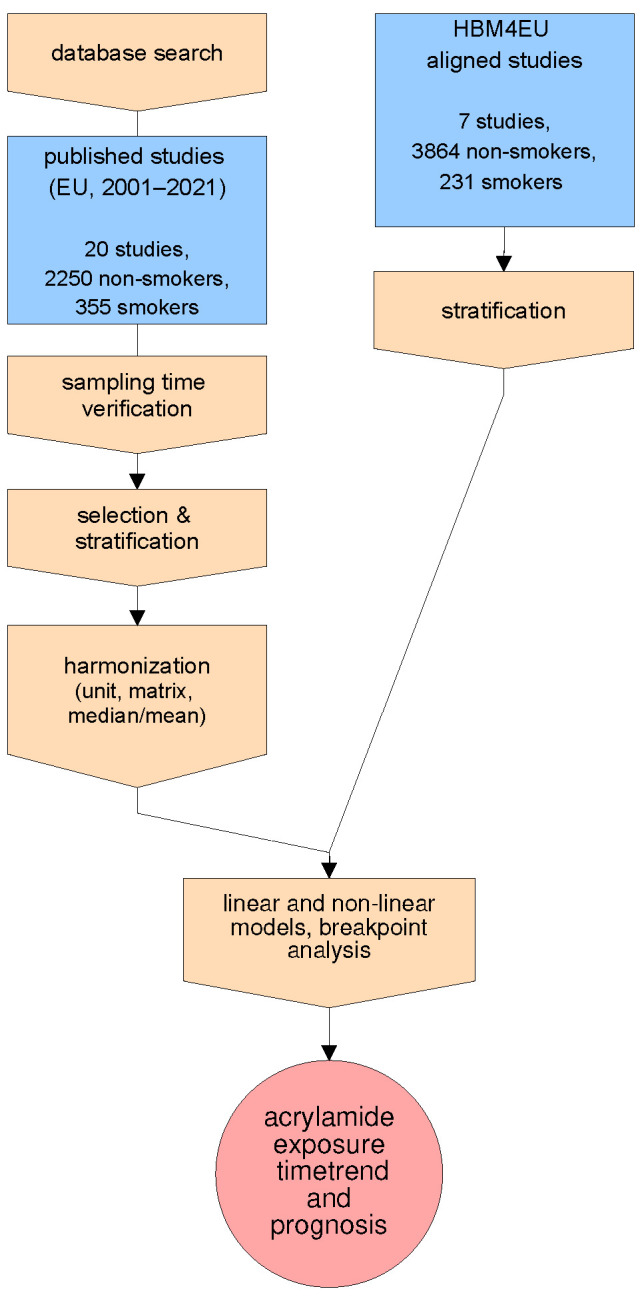
Flowchart of study design.

As the time period covered by dedicated European biomonitoring studies is currently still limited, data from HBM4EU Aligned Studies (collected between 2014–2021) have been complemented by previously published studies (2001–2021) to obtain a sufficient number of biomonitoring records for Europe. Thus, this study relies on previously published papers as well as HBM4EU Aligned Studies (Figure 1). Aggregated results from both groups of sources were harmonized and integrated as well as utilized in separate analyses and validation processes if applicable.

Since previously published data mainly cover the years 2001 to 2015, recent European biomonitoring studies were integrated into the analysis to represent the period after 2015. The HBM4EU Aligned Studies aimed at collecting HBM samples and generating comparable data to derive current internal exposure levels representative of the European population/citizens across a geographic spread [33]. However, it has to be mentioned that in the case of acrylamide, exposure values from different European countries collected from scientific literature and by HBM4EU Aligned Studies should not be generally considered as a representative national or regional cross-section, but are rather understood as values describing exemplary samples collected in EU member states (i.e., country/population size specific weights not applied in analysis).

### 2.1. Collection and Integration of Published Data on European Acrylamide Biomarker Levels

To obtain a time trend of acrylamide exposure in European populations for the period between 2001 and 2015, we collected biomonitoring data from published scientific publications engaged in the biomonitoring of acrylamide. We searched web-based data sources (National Center for Biotechnology Information (NCBI), Web of Science) for European studies providing human biomonitoring data of either AAVal and GAVal in blood or AAMA and GAMA in urine, as these are the most commonly examined biomarkers of acrylamide exposure. For the initial database search, we used the search term: “acrylamide AND 2000/01/01:2022/01/01[dp] AND (“AAMA” OR “GAMA” OR glycidamide OR “mercapturic acids” OR biomonitoring OR biomarkers OR “haemoglobin adducts” OR blood OR urine OR adducts OR “AAVal” OR “GAVal”)”. The obtained list (1915 search results) was then revised for non-European studies, reviews, methodological papers, and acrylamide studies not providing human biomonitoring data that have been excluded. However, our list of European biomonitoring studies is not solely based on online searches. References within retrieved publications were searched and, according to studies, have also been added to the database, including a thesis not shown in PubMed [34].

We did not include biomonitoring studies of populations that have not been stratified for smokers and non-smokers (mixed populations), as these groups differ significantly in acrylamide levels [35]. Reports based on less than 6 individual participants were not included and the populations of considered publications ranged from N = 6 to N = 857 (The minimal sample number of 6 was suggested by the International Council on Harmonisation of Technical Requirements for Registration of Pharmaceuticals for Human Use (ICH) for validation of means from analytical studies [36]). An overview is shown in Table 1. Where no year of sampling was provided in the publication, the authors of the papers were contacted to obtain this information. For some early reports, missing information about the specific sampling year, as well as potential differences to current technical and methodological standards, limited comparability and therefore publications published before 2000 have not been included. Further, studies that did collect samples without providing yearly means or sampling year (see Table 1: 1(LH), 2(MOS), 3(AV1), 6(SC)) have not been included in the analysis, as a temporal correlation of mean biomarker levels on a yearly scale cannot be obtained from these studies. One study (16, TDS) collected samples over a three-year period (2007–2009) without providing yearly means. To include this study in the analysis, we set the sampling year to 2008, accepting a maximal possible deviation of 1 year in time assignment. Of the previously published studies, 9 provided urine metabolite levels, two of them based on 24 h-urine samples 5(MU), 20(GF), one on 12 h-urine 19(KG), five on spot urine 9(MB), 13(UH), 15(EKK), 11(EH), 22(GS), and one on morning urine 17(HM). We observed that acrylamide levels based on 24 h-urine measurements in µg/L were not comparable to spot urine samples, while creatinine corrected values from the same studies appeared to be within the same range, indicating a clear influence of sampling type on urine dilution that has already been described [37]. However, as creatinine corrected values were not available for the majority of published studies it was not possible to include reports based on 24 h-sampling of urine in long-term time-trend analysis.

A detailed comparative description of methods for most of the included published studies has been presented in a paper by Albiach-Delgado et al. (2022) [56]. Despite existing methodological differences in biomarker quantification within European laboratories, results from these sources have been considered comparable and were reviewed within multiple publications [42,46,55,57].

### 2.2. Datasets Provided by HBM4EU Aligned Studies

Acrylamide HBM values of previously published studies have been complemented by data collected in dedicated European studies (HBM4EU Aligned Studies: Participating studies collaborate on aligning human biomonitoring studies in the general population with combined financing from countries and HBM4EU). Among other pollutants, acrylamide was selected to be investigated as a contaminant of concern and specific research questions were formulated to be answered by human biomonitoring surveys. European countries/studies providing acrylamide data were from Italy (Section of Hygiene and Epidemiology within the Department of Medical and Biological Sciences of the University of Udine, EPIUD: Northern Adriatic cohort II, NAC), Portugal (Institute of Health Dr. Ricardo Jorge, INSA: Exposure of the Portuguese Population to Environmental Chemicals: a study nested in INSEF, INSEF-ExpoQuim), Germany (German Environment Agency, UBA: German Environmental Survey 2014–2017, GerES V, France (Agence Nationale De Sante Publique, ANSP: Etude de santé sur l’environnement, la biosurveillance, l’activité physique et la nutrition, ESTEBAN), Luxembourg (Laboratoire national de santé, LNS: Observation of cardiovascular risk factors in Luxembourg, Oriscav-Lux2), Iceland (University of Iceland, UI: Icelandic National Dietary Survey Diet-HBM) and Norway (Norwegian Institute of Public Health, NIPH: Norwegian Environmental Biobank II, NEB II, NOR). Extended datasets were provided based on a bilateral agreement between the Medical University of Vienna and UBA, GerES V.

As we aimed to compare these studies to published results reporting average exposure values, we utilized HBM4EU Aligned Studies results in an aggregated form (arithmetic mean), despite these studies, were providing individual data. Arithmetic mean and biomarker concentrations in µg/L are used in this analysis for comparison, despite the availability of geometric means, medians, and creatinine corrected biomarker concentrations in HBM4EU studies, as arithmetric means in µg/L are reported by the majority of published papers. To achieve comparability within a maximal number of studies, common units and statistical parameters have been utilized from literature as well as HBM4EU studies.

UBA (GerES V, Germany) provided a set of individual data, comprising 2218 children and teenagers (non-smokers) with a mean age of 10.3 years. EPIUD (NAC II, Italy) provided 297 individual data covering a mean age of 7.1 years, and from NIPH (NEB II, Norway) 289 individual data were included, describing a group with a mean age of 9.8 years. INSA (INSEF-ExpoQuim, Portugal) provided 214 datasets of non-smokers (80 smokers) with a mean age of 34.7 years, UI (Diet HBM Iceland) 182 datasets of non-smokers (18 smokers) with a mean age of 31 years and LNS (Oriscav-Lux2, Luxembourg) 169 non-smoker datasets (32 smokers) with a mean age of 33.5 years. ANSP (ESTEBAN, France) provided data on adults and children with 197 non-smoking adults (mean age 32.4 years), 300 non-smoking children (mean age 8.78 years) and 101 smokers (mean age 32.4 years). Basic properties of the HBM4EU Aligned Studies have been outlined in Table 2.

Comparability and quality of biomarker quantification were under the supervision of the HBM4EU Quality Assurance/Quality Control program [58], see also Deliverable 9.4, The Quality Assurance/Quality Control Scheme in the HBM4EU project [59]. In the applied QA/QC scheme, selected expert laboratories participated in three rounds of interlaboratory comparison investigations. The results were used to identify laboratories capable of generating consistent and comparable results for sample analysis in the frame of HBM4EU. Some datasets (ANSP ESTEBAN (children), UBA GerES V, EPIUD NACII, NIPH NEBII) were generated before the establishment of the HBM4EU QA/QC program and comparability is therefore not guaranteed by the HBM4EU Quality Assurance Unit (QAU).

### 2.3. Data Stratification

We stratified data for the analysis for non-smokers and smokers, as acrylamide exposure in these groups was investigated in all included European published studies separately (see Table 1). Regional differences were considered and highlighted.

Another potentially important confounding factor in human biomonitoring is the sex of the participants [60]. Physiological and/or behavioral differences between male and female participants may affect biomonitoring outcomes and, according to the prevalence of the sexes in sampling populations, can potentially cause bias when comparing population means.The sex of the participant has therefore been recorded in HBM4EU Aligned Studies and has also been reported in previously published studies. We did not stratify for sex as a clear dependence of urinary acrylamide biomarkers has not been shown so far. However, we are aware that related factors, like individual urine density levels, have been shown to be specific for sex [37]. This limitation is however regarded not substantial for the given database, as contributing studies are predominantly based on mixed populations, including HBM4EU Aligned Studies. Among previously published biomonitoring studies, only two have been performed on women only (maternal blood, post-partum) comprising a total number of 159 participants and one in men only (92 participants). We did also not stratify for age groups, as the resulting fragmentation of available data would prohibit a conclusive time-trend analysis but alternatively decided to consider information on age as well as the deviation from 50% male/female populations in form of covariates in multiple regression analysis.

### 2.4. Data Harmonization

Results reported in previously published studies were used in this analysis, like findings by HBM4EU Aligned Studies, without modification if possible. However, the major challenge for an analysis based on a comparison of independent published and unpublished studies is the heterogeneity of sampling conditions, analytical methods, reported units, and other parameters. We observed heterogeneity in the sampling matrix, population parameters, like average age, as well as in the geographical distribution of sample origin. But also different units of quantification have been used in reports as well as different statistical methods to describe the population exposure (mean, median, standard error, quartiles).

Large populations investigated by HBM4EU Aligned Studies were providing urine biomarkers of acrylamide (AAMA and GAMA), while many previously published studies were based on hemoglobin adduct concentrations. Thus we estimated mean urine biomarker levels from available blood adduct concentrations using a method outlined below (Section 2.4.2, trans-matrix estimates). Reliability of these trans-matrix estimates are limited by an added level of uncertainty and we therefore performed multiple linear regressions with datasets including and excluding these values.

**Figure 2:**
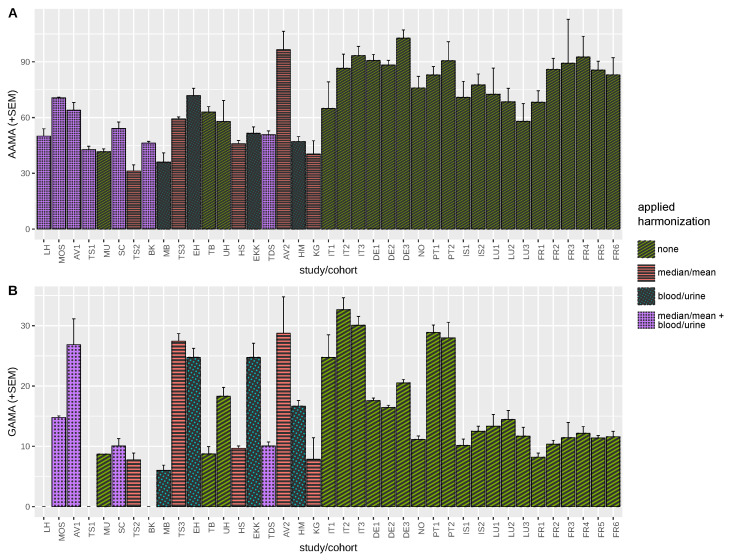
Harmonized overview of mean reported acrylamide biomonitoring levels based on previously published literature and HBM4EU Aligned Studies (2000–2021). Mean/estimated mean (+standard error) for AAMA (µg/L, (**A**)) and GAMA (µg/L, (**B**)), color/pattern coded for applied harmonization calculation method. Standard error of the mean (SEM) is shown for visual comparison of variance taking into account of differences in study population size. Study index labels (LH-FR6) are shown according to Table 1.

Urine density corrected values were only available for a minority of previously published studies and time-trend analysis had, therefore, relied on µg/L-units without compensation for creatinine concentrations. Exposure level means are compared, as the majority of literature reports mean values, and estimating medians from mean values is not warranted. If only medians were provided, means were estimated using data range or quartiles if available (Section 2.4.1). Figure 2 shows a graphical representation of available studies 2000–2021 based on first-morning or spot urine (before exclusion of studies due to missing year of sampling), standard errors of the mean (comparable among different population sizes), and the harmonization steps applied to results for AAMA and GAMA.

#### 2.4.1. Estimation of Means from Medians and Ranges or Quartiles

For HBM4EU Aligned Studies providing individual data, it was possible to calculate mean values and according to variances. However, for the extended time-trend analysis, means had to be determined also for previously published studies, which were often reporting medians and counts or quartile ranges. The estimation of means from provided medians is complicated by the fact that, in most cases, we could not assume the underlying data to be normally distributed. We thus applied mathematical methods suggested by Hozo et al. and Wan et al. [61,62] to obtain approximations for means and standard deviations using the following equations:

(1) $${\bar{X}}_{1}\approx \frac{a+2m+b}{4},{\bar{X}}_{2}\approx \frac{q1+m+q3}{3}$$

(2) $$S_{1}\approx \frac{1}{\sqrt{12}}{\left[\sqrt{b-a}+\frac{a-2m+b^{2}}{4}\right]}^{12}$$

(3) $$S_{2}\approx \frac{b-a}{4}$$

(4) $$S_{3}\approx \frac{b-a}{6}$$

*X* = arithmetic mean, *m* = median, *a* + *b* = maximal and minimal values, *q*1, *q*3 = first and third quartile. Estimation of standard deviation from median and data ranges (1). S_1,2_ = standard deviation, estimation for n≤ 15 (2), 15 < n≤ 70 (3) and n> 70 (4).

#### 2.4.2. Calculations of Estimates for Urine Acrylamide Metabolite Concentrations from Available Blood Adduct Levels (Trans-Matrix Estimates)

For recent years (2014–2021) mean biomarker levels are reported from a large number of urine samples (3865), while the years 2001–2013 are represented by only 337 urine samples. To address this imbalance in sample numbers, we aimed to add 1043 blood samples collected before 2012 to our analysis. To achieve this, means/medians from one matrix (Hb-adduct levels) had to be used to calculate an estimate for the other (urine metabolite concentrations).

As acrylamide levels from both matrices are generally considered to be valid representatives of the underlying acrylamide exposure, indicators from both matrices are not independent, but represent a given exposure level influenced by different forms of elimination kinetics [22]. Therefore, time between acrylamide exposure and its representation by the according biomarker level is different for blood and urine and dual matrix sampling in individuals at one point in time is not expected to show strictly correlating values due to different corresponding intake doses.

However, results from studies providing data from both matrices and in participants with constant exposure, as typically observed in smokers, show that the relationship is indeed rather linear, at least within the most relevant range of observations. This shows that a stable relationship between AAVal and AAMA can be observed in simultaneously sampled probes of individuals in the very special situation of constant exposure, as both biomarkers, regardless of actual sampling time, are resulting from very similar intake levels. The constant exposure is however only required for the correlation of simultaneously collected samples, while the obtained factor may then be used to estimate urine from blood levels that reflect samples separated in time. The mathematical representation of this linear relationship can be taken from the equation describing the coefficients of the linear model (between AAVal and AAMA and between GAVal and GAMA) of a predominantly smoking population. The obtained ratio of acrylamide to glycidamide metabolite may then be used for estimation of biomarker levels in non-smokers, considering that the time lag between the actual exposure and the accordingly observed biomarker level will effectively not affect the assignment of the year of exposure.

As the time-trend analysis is based on mean values determined at the according year of sampling, individual toxicokinetic differences in elimination time of non-smokers will not significantly affect temporal assignment and the urine/blood levels relationship derived from smoking individuals may thus be applied to obtain a valid estimation for the given time resolution of one year.

For the estimation of the conversion factor, a constant exposure level is required, as simultaneous collection of blood and urine will then reflect the same exposure independent of the time of sampling. Constant exposure levels are however not a general prerequisite for the application of the factor, because the maximal time lag (about two weeks) between corresponding urine and blood levels is not relevant in the current analysis.

**Figure 3:**
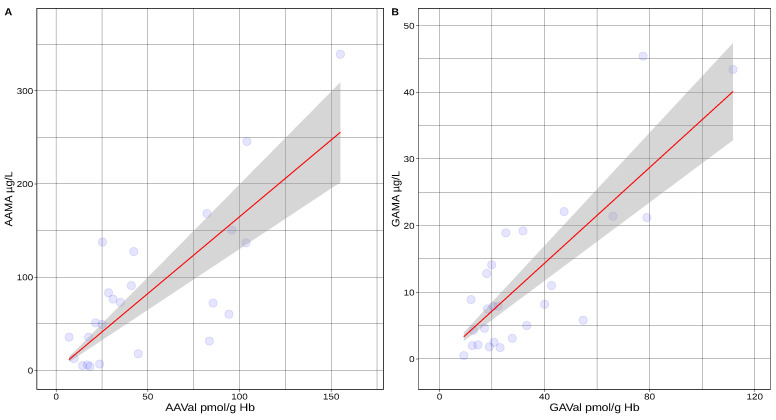
Linear model of corresponding acrylamide biomarkers in blood and urine matrix. Regression of AAMA vs. AAVal ((**A**), AAMA (µg/L) = 1.64 × AAVal, pmol/g Hb, *p* < 0.0001) and GAMA and GAVal ((**B**), GAMA (µg/L) = 0.35 × GaVal, pmol/g Hb, *p* < 0.0001) in individuals (data kindly provided by T. Schettgen, M.B. Boettcher, J. Angerer). Red = regression line, blue = data points, grey = 95% confidence interval.

Data of individual dual-matrix determinations of acrylamide levels were provided by T. Schettgen (unpublished results). This dual-matrix study is including biomarker levels of a majority of smokers (13 of a total of 24 participants), with a daily consumption ranging from 2 up to 10 cigarettes per day (Figure 3). The experimental proof of the concept as outlined above is provided by the linear correlation of biomarkers from both matrices.

We assumed that if AAVal/GAVal levels equal zero in a hypothetical no-exposure scenario, also AAMA/GAMA levels were expected to be at 0 µg/L or at least very close to zero and did thus assume no intercept. The urine AAMA and GAMA concentrations (in µg/L) could thus be estimated as:

(5) $$AAMA_{µg/L}\approx 1.64\times AAVal_{\mathrm{pmol}/g},GAMA_{µg/L}\approx 0.35\times GAVal_{\mathrm{pmol}/g}$$

**Figure A1:**
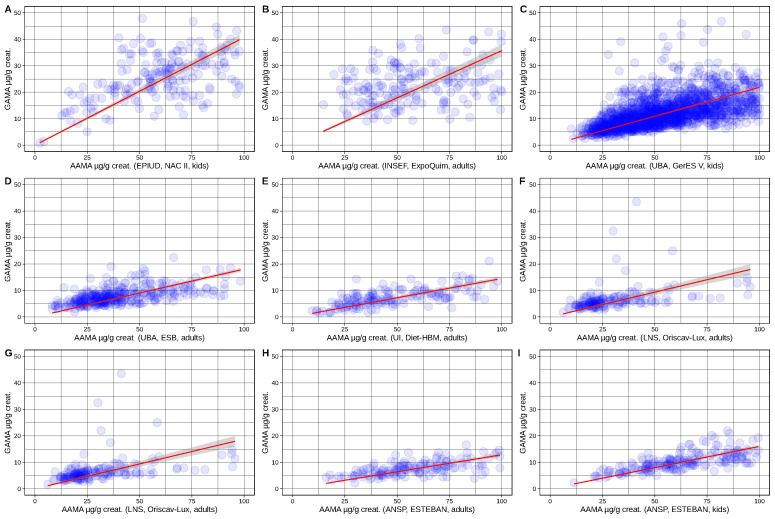
Plots of AAMA vs. GAMA (in µg/L), in 9 different European populations ((**A**–**I**), individual data) as indicated. Linear fit in red, gray = 95%. AAMA to GAMA correlation is linear within the range of observations and the slope is depending on region/country, but not age, as indicated by data from France based on kids and adults (**H**,**I**). Higher GAMA-to-AAMA level ratio is reported from Italy (EPIUD, (**A**)) and Porugal (INSEF, (**B**)), low ratio from France (**H**,**I**).

The above approximation implies that there is a linear correlation between AAMA and GAMA levels, as the ratio for conversion factors remains constant. Our results obtained from individual data of HBM4EU Aligned Studies show that this is indeed the case (Figure A1). However, the slope of the AAMA-to-GAMA level correlation was found to be different in investigated populations of Europe. These observed differences are very likely due to variability in metabolism. GAMA conversion factors can therefore only be applied within a (regional) population.

#### 2.4.3. Influence of Age on Acrylamide Time-Trends

Bjellaas et al. (2007) [47] showed, that AAVal and GAVal concentrations (pg/g Hb) of non-smokers are dependent on age, with higher exposure values observed at younger ages and lower in older persons. As acrylamide biomonitoring studies were performed in populations comprising either children or adults, the mean age at sampling time may have introduced an influencing factor to potential time-trends and should thus be considered. Therefore, in addition to aggregated data presented here, individual data from HBM4EU-aligned and partner studies were analyzed to quantify the correlation of urine acrylamide indicators and age by linear regression. The analysis revealed that, in accordance to Bjelleaas et al., a clear negative correlation was found in AAMA and GAMA levels on age when compensated for urine density (in µg/g creatinine) (not shown). For values measured in units of µg/L, the association of age and urine acrylamide metabolite level was not significant for AAMA in individual data. We, therefore, did not compensate for age differences in graphical depictions, but controlled for the reported mean age of study populations in multiple linear regressions to minimize this influence on statistical outcomes.

#### 2.4.4. Influence of Sex on Acrylamide Time-Trends

An analysis of individual data from HBM4EU Aligned Studies did not reveal a significant difference in acrylamide biomarker levels (in µg/g creatinine) in dependence on sex. However, there are indications that mean urine density may be different in sexes and as acrylamide biomarker levels are here reported in µg/L, urine density may finally affect these values. Observed trends could thus hypothetically be affected by differences in the distribution of sexes between included study populations. HBM4EU Aligned Studies have been performed in populations comprising both male and female participants and previously published studies are based on populations with very variable distribution of sexes. To test for a possible influence on observed trends due to the distribution of sexes in underlying populations, we used the percent values of males for each study as covariates in multiple regression.

#### 2.4.5. Statistics

To take into account the large difference in study sample (N) numbers, we choose a model weighted for population size for (multiple) regression analysis. After applying harmonization steps (Figure 1 and Figure 2), we statistically analyzed the time-trend of mean AAMA and GAMA biomarker levels. The mean values of AAMA and GAMA were followed using a yearly scale, which was the highest resolution of time that could be determined for all studies providing aggregated data. For obtaining information about the actual time-trend, a simple weighted linear model was applied to study mean values that allow for extrapolation.

As the combined time trend analysis is based on arithmetic means from different sources, we expected a high degree of variability in variance (standard deviation) associated with each mean. Study population size was therefore used as the weighting factor in the time-trend linear model, under the assumption that studies based on larger populations provide a higher precision in determining the mean. To test if mitigation measures could have influenced the observed acrylamide exposure timeline, potential changes in the slope of the time-trend had to be visualized and statistically tested. Therefore, a non-linear fit using a local regression (loess, locally weighted scatter-plot smoothing) was also applied to visualize non-linear trends. To statistically test for possible temporal changes in the slope of the trend (breakpoints) a regression model with segmented relationships (R: segmented.lm) was utilized. The distribution of regression residuals of the linear model has also been visually inspected for heteroscedasticity. Normal distribution was tested by the Shapiro-Wilk test (R: shapiro.test) and Q-Q-plots (R: qqnorm, qqline).

Statistical calculations have been performed using R [63].

## 3. Results

Significant variability was observed between individual exposure biomarkers levels for acrylamide in European populations. This variability may be due to multiple reasons, including differences in individual diet/food and/or smoking habits of participants as well as in mean age of participants and metabolism and, to a much smaller degree, the differences in detection limits and accuracy of the methods used for measurement.

**Figure 4:**
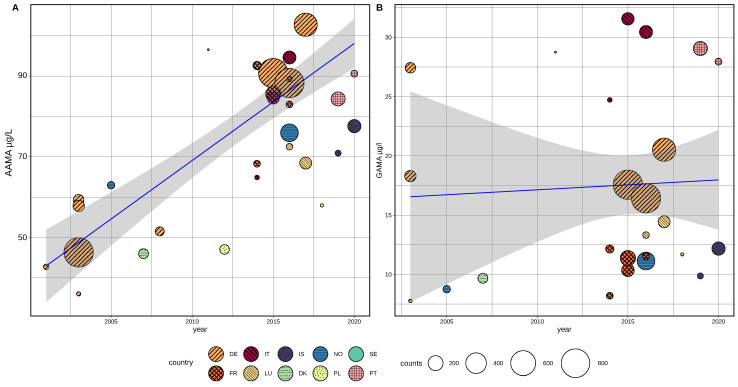
Linear model (regression) of harmonized means of acrylamide biomarkers AAMA and GAMA (non-smokers) as reported by European published papers and HBM4EU Aligned Studies (AAMA 30 studies, GAMA 29 studies). Regional distribution is indicated by colors and relative population size is shown by the size of the circles indicating single surveys. (**A**): AAMA in µg/L, (**B**): GAMA in µg/L. Gray: 95%-confidence interval. Regression line in blue.

A linear regression model based on mean values from non-smokers, weighted for study population size and using population age and sex representation as covariates, indicate an increase of AAMA levels within the time period of 2001–2017 (Figure 4A). Within a period of 13 years (2003–2016) the AAMA levels were observed to increase by about 50%.

Multiple weighted regression adding mean population age as a variable showed a significant increase (estimate: +2.09 µg/L per year, *p* < 0.0001) for AAMA and a slightly negative trend for age (estimate: −0.49 µg/L per year, *p* < 0.001). Differences in sex distribution (% of male samples) in study populations were used as another confounding factor in multiple regression and was found to be not significant for AAMA and GAMA. Due to the given study population sizes, the weighted linear regression trend for AAMA is predominantly determined by the large population study by Kütting et al. (sampling 2003) and samples from the HBM4EU aligned study GerES V (2015–2017), both based on German populations.

The above analysis includes yearly means for AAMA determined by estimation of urine concentrations based on measured blood levels, including a total of 30 yearly means based on 5245 samples. As the applied method of trans-matrix estimation is introducing a level of uncertainty to the analysis, we also performed the same analysis solely based on means from urine samples (25 yearly means based on 4202 samples, Table 3). Multiple weighted regression adding mean population age and sex balance as covariates showed a significant increase (estimate: +1.91 µg/L per year, *p* = 0.0004) for AAMA and a slightly negative trend for age (estimate: −0.45 µg/L per year(age), *p* = 0.034) for age. Inclusion of estimated values (blood-to-urine), representing 1043 samples had only a minor impact on trend-line slope, but improved significance from *p* = 0.0004 to 0.0001 for AAMA and from *p* = 0.004 to 0.00035 for age. For GAMA exposure levels, the time-trend was found to be not significant (tested by ANOVA + weighted regression) in the linear model based on published studies (Figure 4B).

Despite being mainly based on spot/morning urine samples, the overall trend for AAMA levels appears to be relatively consistent for different regional populations, as indicated by generally lower levels at earlier and higher sample concentrations at later timepoints.

For mean GAMA levels, no significant time-trend could be found in European studies 2000–2021. However, data from aligned studies based on individual data indicate that the lack of significance is mainly due to a higher variability in GAMA means, possibly driven by differences between regions and related CYP2E1 (cytochrome P450 2E1, see also discussion Section 1) treating polymorphism prevalence. Other than AAMA concentrations, individual GAMA levels depend on enzymatic activity of CYP2E1, which has been shown to be affected by polymorphisms resulting in regional variable acrylamide metabolism [64,65,66,67]. Figure 4B shows that GAMA levels from south European countries (Italy, Portugal) are higher than values reported by northern countries (Norway, Iceland), and accordingly GAMA levels are higher at similar AAMA concentrations in southern countries (Figure A1). However, as indicated by results shown in Figure A1, GAMA is well correlated with AAMA within regional populations, while significant differences appear between studies. This finding implies that an observed rise over time in mean AAMA levels will very likely also result in a rise in mean GAMA concentrations, but as GAMA also depends on regional determinants, the trend may be obscured in a combined, multi-regional overview. The absence of a time-trend in GAMA levels can therefore be explained by regional differences and a lower number of data points that is due to the fact that regional differences can not be taken into account in data harmonization.

Time correlated comparison of harmonized exposure values indicates the lowest mean GAMA levels (≤13 µg/L) in samples from countries in northern and central/western Europe (France, Denmark, Norway, and Iceland), higher levels (13–23 µg/L) were mainly reported in central European countries (Germany and Luxembourg) and highest (>23 µg/L) mean exposure values in samples from southern countries of Europe (Italy and Portugal), as best visible for the well documented time segment 2010–2020 (Figure 4B).

**Figure 5:**
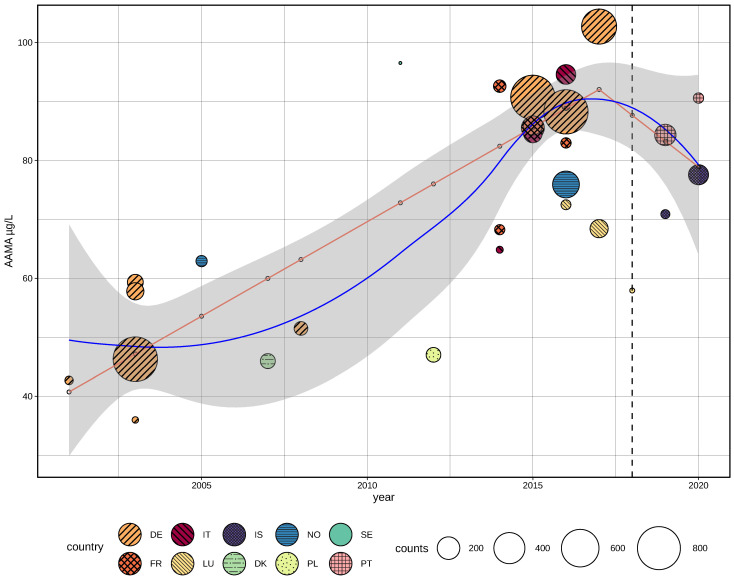
Locally fitted scatter-plot fit (LOESS) for AAMA time-trend data (harmonized, non-smokers). Study means represented by country color-coded dots with size dependent on count number and 95%-confidence interval (gray). LOESS fit line in blue. The red line shows the trend of a multi-segmented regression analysis (significant break-point detected in 2017, *p* < 0.001). Dashed line indicating the time-point of Commission Regulation 2017/2158 put in force (2018).

As potential changes in acrylamide exposure due to previous mitigation measures were of central interest in this analysis, we also applied a non-linear fit (locally fitted scatter-plot fitting, LOESS) to the time-trend data from aggregated sources to visualize an alternative non-linear trend line. The non-linear fit indicates a rather stable phase until 2007, a steep increase until 2017 and a possible flattening for 2018–2020 with a reduced confidence close to more recent times due to insufficient data (Figure 5, blue line). Consequently, break-point-analysis in form of a multi-segmented regression was performed, with the aim to detect potential changes in the slope of the trend line (Figure 5, red line).

A regression model with segmented relationships (breakpoint analysis, including estimated values) revealed a significant change in the time-dependent slope of AAMA in 2017. The LOESS curve also peaks in 2017, one year before the implementation of mitigation measures by the EU (Commission Regulation 2017/2158 [30], dashed line).

**Figure 6:**
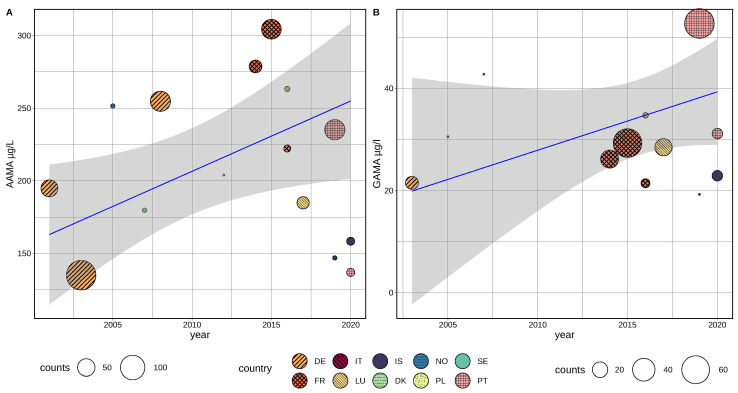
Linear model (regression) of harmonized means of acrylamide biomarkers AAMA and GAMA in smokers as reported by European published papers and HBM4EU Aligned Studies. Study means represented by country based color-coded dots with size dependent on the corresponding sample number. Population size is represented by the size of the circles indicating single surveys. (**A**): AAMA in µg/L, (**B**): GAMA in µg/L. Gray: 95%-confidence interval. Regression line is in blue.

We also investigated the AAMA time-trend for smokers in Europe since 2001 (Figure 6). Smokers are known to display significantly higher exposure values for both AAMA and GAMA, but number and populations of previously reported studies in smokers were much smaller than in non-smokers. However, HBM4EU Aligned Studies comprising biomarker levels in adults did provide recent exposure levels of smokers, allowing for analysis of the time-trend after harmonization steps as described in the methods section. The linear time-trend model of AAMA levels in smokers shows a significant rise in a generalized least squares model and ANOVA (*p* < 0.05). For GAMA, no significant trend could be found.

## 4. Discussion

### 4.1. Time-Trend of Acrylamide Exposure in Europe 2001–2021

Our combined analysis of human biomonitoring results for acrylamide from retrospective and current data based on HBM4EU Aligned Studies indicates an increase of exposure levels within the period 2003–2017 in non-smokers. Due to the heterogeneity of available human biomonitoring data sources, uncertainties remain regarding the slope of the long-term trend and thus limiting the possibility of exactly predicting further development (in hypothetical absence of additional measures).

We found a very similar increase (slope) in AAMA (µg/L) in a database containing mean values estimated from blood and urine samples as well as in an analysis solely based on urine. Means based on samples from urine were available for recent years (2014–2021), while only a few publications reported means from urine samples between 2000 and 2014. By estimation of mean urine concentrations based on blood samples, roughly 1/5 of the total number of samples (1043 of 5245) could be added for the time period 2000–2014. The addition of estimated means from blood levels did only improve the significance of solely urine-based analysis. The applied method of estimation of urine from blood metabolite concentrations is rather crude and derived results have to be reviewed with caution. However, another dual-matrix study was performed by Urban et al. (2006) [35], provides aggregated data for a rough validation of AAVal to AAMA estimations. Based on data of 60 smokers, a median of 79.1 pmol/g Hb AAVal corresponded with 107.3 ng/mL AAMA. Using the above approximation, 129.7 ng/mL were estimated, indicating an error of +20.87% for smokers. Application of this simple method to data of the same study based on non-smokers, with a reported a median of 26.8 pmol/g Hb, results in an estimated value of 43.9 ng/mL at a measured median of 41.6 ng/mL and an estimation error of +5.52% (the study by Urban et. al. did collect 24 h-urine, while the estimation is based on spot urine). The third European study presenting acrylamide biomarker data from blood and urine in the same population, E.C. Hartmann et al. (2008) [46], does not provide mean levels, but medians and ranges. We can use the method by Hozo et al. [61] to estimate a mean value for AAMA of 64.5 µg/L (based on median 29, range LOD-229) and AAVal of 36 pmol/g (based on median 60, range 15–71). Using our method of calculating urine from blood levels we obtain 1.64 × 36 = 59.04 µg/L, an error of +8.5%. These calculations suggest, that the error in estimation using our method is below 10% for AAMA in non-smokers.

As the overall time-trend is mainly determined by a large number and size of recent studies and a relatively small total number of samples collected earlier, our analysis shows that the inclusion of 1045 blood samples from that earlier period confirms the observed trend. In other words, the large number (857 non-smokers) of blood-based samples from by Kuetting et al. (sampled in 2003) is located exactly at the point of the time-trend curve, where we expect them to be, based on the trans-matrix estimation.

The observed increase in mean exposure since 2001 of about 40% for AAMA cannot be considered minor. This is emphasized when comparing reported biomonitoring data with the proposed Biomonitoring Equivalent (BE) values for AAMA, concentration levels that are regarded as consistent with existing health-based guidance values [68] and were determined with 13 µg/L (16 µg/g creatinine, overall population) for AAMA.

These values have been calculated for different age groups (children <13 years, adolescents 13–18 years, adults >19 years) based on doses determined in animal experiments and on an US risk assessment [69], which concluded that the area under the serum curves (AUC) for acrylamide and glycidamide represents appropriate dose metrics for neurological and tumor responses. Available BE for acrylamide is therefore limited by a level of inherent uncertainty, indicating the need for updated European human biomonitoring guideline values.

A rough visual extrapolation of the trend in Figure 4 indicates that current spot urine levels, at least in some populations of Europe, exceed mean levels of 70 µg/L for AAMA (according to the lower level of confidence interval at the end of the currently available timeline).

A non-linear fit, as shown in Figure 5, may provide a more detailed view of acrylamide exposure development although it does not allow for a simple statistical evaluation. We, therefore, applied a multi-segmented regression model to detect significant changes in the slope of the trend (breakpoint analysis) and to determine possible changes in mean exposure development, as anticipated after the application of mitigation measures. The breakpoint-analysis did point out a significant change in the slope of the time-trend for the year 2017. The mitigation measure with the highest probability of resulting in detectable changes among regulative actions applied so-far was Commission Regulation 2017/2158, that was put in force in April 2018 (Figure 5, dashed line). The determination of a breakpoint one year earlier may be simply due to the fact, that very little data for the year 2018 itself were available for this study. However, in contrast to the time period covered before 2018, very recent studies have been performed in adults only and we thus can only speculate if the same trend could be observed in all ages since 2018. AAMA levels of smokers (in µg/L) have also been found to be rising since 2001, while a similar rise in GAMA was not statistically significant.

We can here only speculate about the reason for the apparent rise in AAMA levels since 2001. As dietary intake represents the main form of uptake in the general non-smoking population [70], nutrition-related exposure is the most likely source responsible for an increase in urinary AAMA levels in non-smokers. A detailed questionnaire-based analysis of exposure determinants for HBM4EU-aligned studies is performed separately and will provide more information on main sources of acrylamide exposure for contributing study populations.

Our analysis indicates that mean AAMA levels did rise in all investigated age groups, demonstrated by the significance of linear regression after compensation for age differences and also visible in Figure 4, as samples taken after 2012 are generally higher than values reported between 2001 and 2012, irrespective of age. Data from France (ANSP, ESTEBAN), collected in 208 children and 138 adults (in 2015) do not show a significant difference in mean AAMA levels in µg/L (children 85.52 µg/L, adults 85.94 µg/L).

The rise in AAMA observed in smokers is remarkable, as the primary source of exposure is different in this group, and the relative contribution of dietary acrylamide is limited. The increase over time in smokers is, however, when compared to non-smokers, clearly smaller. Considering the higher variability of exposure values in smokers, the observed rise in smoking participants could be explained by an increase from dietary sources that is complemented by a, possibly even constant, exposure to cigarette smoking.

### 4.2. Regional Trends

European regional differences in time-trends of acrylamide are indicated and appear to be smaller for AAMA than GAMA. In Figure 4B we can see that GAMA exposure levels from Norway, Iceland, France, Poland, and Denmark are generally lower than those from Germany (orange), but the time trend appears to run in parallel to the main trend-line for this group. We lack sufficient retrospective data to also see a trend for countries of southern Europe, but a similar time-course starting at slightly higher levels, as compared to data from Germany, can be observed for data from this region (Portugal and Italy).

The regional differences observed in AAMA-to-GAMA (Figure A1) correlation slopes are very likely due to regional variability in acrylamide metabolism efficiency. Polymorphisms of CYP2E1 have been shown to determine the efficiency of formation of GA from acrylamide [71]. While differences in excretion kinetics in individuals prohibit the detection of such polymorphisms in individuals [72,73], ethnicity-based clustering of polymorphisms may be well reflected in aggregated data, as it has been shown for populations in the US [74] and eastern Europe [75]. Such ethnicity based regional differences in acrylamide oxidation are likely to affect urine GAMA but lower AAMA levels.

As carcinogenicity of acrylamide is mainly induced by GAMA, genetic predisposition may contribute to a large part to individual cancer risk associated with acrylamide exposition. As AAMA-to-GAMA correlations suggest, the same increase of exposure to acrylamide may result in a significantly different GAMA-linked cancer risk in different regions of Europe. These regional differences appear not to depend on age, as samples from adults and children of the same regional population show similar AAMA-to-GAMA correlation slopes (Figure A1G,H) while samples from adults and children are very different across European regions (Figure A1A,B,G,H). This also indicates that a possibly not fully developed metabolism in kids does not account for the observed differences.

### 4.3. Limitations

In this study, we aimed to obtain an overview of the time trend of acrylamide exposure in Europe, by integrating all available data sources and limitations of this integrative approach are mainly related to data harmonization. For obtaining sufficient data points for time-tend analysis, we had to introduce a level of uncertainty by estimating urinary from blood biomarker levels and mean levels from medians. Applied calculations for estimations are however based on comprehensible considerations. Further, this study is complemented by our second paper based on HBM4EU studies only (Trends of Exposure to Acrylamide as Measured by Urinary Biomarkers Levels within the HBM4EU Biomonitoring Aligned Studies (2000–2021) [76]), where harmonization steps were not required and which results may be compared within the overlapping time period.

We are aware that a comparison of a urinary human biomonitoring marker, mainly based on spot samples without correction for creatinine is far from optimal in the given context, but rather represents a compromise that had to be accepted in exchange for a sufficient number of data points. As creatinine corrected urine biomarker levels were not provided by all published studies, harmonization steps had to be based on AAMA and GAMA levels. Variability of urine density is generally observed to be substantial in individuals, but for average values of larger populations, acrylamide biomarker levels in µg/L and in µg/g creatinine are typically well correlated, as individual differences in urine density converge around a mean value. It is hypothetically possible, that the observed trend in biomarker concentrations is due to a long-term time-trend of urine density and not of AAMA and GAMA concentrations. A long term trend in urine was indeed already described in the study by Lermen et al. [37]. However, this study, based on samples from Germany, showed that the long-term trend in urine density results in lower specific gravity and creatinine concentrations at higher total urine volume levels in 2016 as compared to 1998. Thus, the trend as described in this paper would very likely lead to an opposite time dependence for AAMA and GAMA levels as observed and indicates that the slope of our analysis may in fact be even slightly steeper than described here. Another consequence of dependence on urine density has been described in the same paper, showing a significantly higher specific gravity and conductivity in men compared with women. Böttcher et al. (2006) [21] indeed showed lower levels of AAMA and GAMA (in µg/L) of women and a concomitant deviation of mean values derived from included single-sex-based studies is possible. However, the total number of data from men and women is, as far as provided, rather equally distributed along the presented timeline and could demonstrate no relevant shift due to distribution differences in participant sex of included studies (Table 3). Differences in the mean age of study populations may affect also the trends of urinary biomarkers, but have been considered as an additional independent variable in statistical analysis (multiple regression).

A significant methodological improvement of AAMA biomonitoring has been introduced by Boettcher and Angerer in 2005 [77], which allowed to discriminate AAMA from acrylonitrile. As acrylonitrile is an environmental pollutant and occupational exposure of this industrially produced compound has to be anticipated, it may contribute to reported exposure levels determined in years before the adaptation of the above-mentioned methods. We observed a higher variability in studies published before 2005. However, occupational exposure and industrial spills that are mainly responsible for acrylonitrile exposure are very likely affecting only relatively small groups of individuals and the higher variability may thus be rather due to other methodological issues.

## 5. Conclusions

Our extended analysis based on previously published literature and recent acrylamide biomonitoring studies shows a significant increase in exposure levels for AAMA in µg/L within the period 2001–2018, already starting at levels well beyond determining biomonitoring equivalents. Efficiency of EU-wide mitigation measures are indicated in this analysis, but due to the rather short time since implementation and other limitations, more analysis in the future is required to confirm the indicated development in recent years.

## Figures and Tables

**Table 1:** Overview of European biomonitoring studies.

Nr.	Author	Pub. Year	Sampl. Year	Matrix	Num. Participants, Non-Smoker/Smoker	Age (Mean, Median, Range) in Years	Sex (Perc. Male)	Rep. Value	AA (ns/s)	GA (ns/s)	Unit
1 [38] X	L. Hagmar et al. (LH)	2005	1991–1996 *	blood	ns 70 s 72	(45–73)	-	median	31 152		pmol/g
2 [39] X	M. Obon-Santacana et al. (MOS)	2016	1992–2000 *	blood	ns 417	M: 58	0	median	43.1	35.4	pmol/g
3 [40] X	Anna C, Vikström et al. (AV1)	2012	1999–2000 *	blood	ns 68	45–73	-	median	39	67	pmol/g
4 [41]	Thomas Schettgen et al. (TS1)	2003	2001	blood	ns 25 s 47	M: 34 (19–59)	88	median	21 85		pmol/g
5 [35] X	Michael Urban et al. (MU)	2006	2002	urine	ns 60 s 60	-	38.3	mean	41.6 107.3	8.7 15.0	µg/L
5 [35] X	Michael Urban et al. (MU)	2006	2002	blood	ns 60 s 60	-	38.3	mean	27.6 81.8	-	pmol/g
6 [42] X	Sylvie Chevolleau et al. (SC)	2007	-	blood	ns 52 s 16	18–77	42.6	mean	27 53	23 34	pmol/L
7 [43]	Thomas Schettgen et al. (TS2)	2004	2003	blood	ns 13 s16	M: 35 (16–67)	23.0	mean	18 80	17 53	pmol/g
8 [44]	Birgitta Kütting et al. (BK)	2009	2003	blood	ns 857 s 148	41.6	46.9	mean	28.2 82.6	-	pmol/g
9 [45]	Melanie Isabell Boettcher et al. (MB)	2005	2003	urine	ns 16 s 13	m: 25.5	31.2	median	29 127	5 19	µg/L
10 [17]	Thomas Schettgen et al. (TS3)	2010	2003	blood	n.s. 92	M: 35 (6–80)	21.7	median	29.9	35.2	pmol/g
11 [46]	Eva C, Hartmann et al. (EH)	2008	2003	urine	ns 91	m: 36	49.4	median	29	7	µg/L
11 [46]	Eva C, Hartmann et al. (EH)	2008	2003	blood	ns 91	m: 36	49.4	median	30	34	pmol/g
12 [47]	Thomas Bjellaas et al. (TB)	2007	2005	blood	ns 44 s 6	m: 45	45.4	mean	38.4 154	19.6 76.6	pmol/g
13 [18]	Ursel Heudorf (UH)	2009	2007	urine	ns 110	5–6	57.2	mean	57.8	18.3	µg/L
14 [48]	Hans von Stedingk et al. (HS)	2011	2007	blood	ns 81 s 6	m: 30	0	mean	28 110	22 102	pmol/g
15 [34]	Eva Katarina Kopp et al. (EKK)	2009	2008	urine	ns 67 s 67	m: 35.5	32.8	median	39 121	9 25	µg/L
16 [49]	Talita Duarte-Salles et al. (TDS)	2013	2007–2009 *	blood	ns 79	m: 30	0	mean	31	23	pmol/g
17 [50]	Anna C Vikström et al. (AV2)	2011	2011	blood	ns 10	m: 46	50	mean	59	72	pmol/g
18 [51]	Hanna Mojska et al. (HM)	2016	2012	urine	ns 78 s 5	m: 30 (20–40)	0	median	18.9 168	6.8 27.7	µg/L
19 [52]	Katharina Goerke et al. (KG)	2019	2015	urine	ns 20	m: 26	50	mean	312	45	nmol/day
20 [53]	Katharina Goempel et al. (KG2)	2017	2015	blood	ns 6	(20–44) ***	100	mean	24.5	17.2	pmol/g
21 [54] X	Gianfranco Frigerio et al. (GF)	2020	2017	urine	ns 38 s 22	m: 46	89.4	median	142 405	1.3 3.2	µg/g creatinine
22 [55] X	Gerda Schwedler et al. (GS)	2021	2015–2017 **	urine	ns 2211 s 48	m: 10.4	51.6	mean	95.33 242.4	-	µg/L

non-smoker: ns, smoker: s, mean: m, median: M, AA: acrylamide biomarker, GA: glycidamide biomarker, X: excluded, * studies collecting samples for multiple years, ** data part of GerES V (excluded because of overlapping data), - not provided, *** age range of exclusion criteria.

**Table 2:** Overview of HBM4EU Aligned Studies and bilateral data sources.

Data Provider	Year of Sampling	No. Participants	Mean Age (Years)	Sex (Perc. Male)	Mean AAMA in µg/L	Mean GAMA in µg/L
EPIUD NAC II (IT1)	2014	ns 18	7.0	0	64.85	24.73
EPIUD NAC II (IT2)	2015	ns 132	7.2	52.3	84.58	31.57
EPIUD NAC II (IT3)	2016	ns 147	7.0	55.1	94.58	30.46
UBA GerES V (DE1)	2015	ns 852	10.3	50.2	90.7	17.57
UBA GerES V (DE2)	2016	ns 849	10.3	47.1	88.24	16.43
UBA GerES V (DE3)	2017	ns 517	10.3	49.7	102.52	20.52
NIPH NEB II (NO)	2016	ns 289	9.8	52.9	75.92	11.13
ANSP ESTEBAN (FR4)	2014	ns 55	8.5	49.1	92.6	12.15
ANSP ESTEBAN (FR5)	2015	ns 208	8.9	52.6	85.52	11.37
ANSP ESTEBAN (FR6)	2016	ns 37	8.9	54.0	82.97	11.59
UI Diet-HBM (IS1)	2019	ns 289 s 6	31.6	53.6	70.88 146.91	9.87 19.24
UI Diet-HBM (IS2)	2020	ns 154 s 12	30.6	41.5	77.57 158.40	12.18 22.89
INSA INSEF-ExpoQuim (PT1)	2019	ns 177 s 67	34.5	39.0	84.32 232.46	29.07 52.07
INSA INSEF-ExpoQuim (PT2)	2020	ns 37 s 12	34.7	40.5	90.85 136.95	27.96 50.62
LNS Oriscav-Lux2 (LU1)	2016	ns 34 s 7	33.3	41.2	72.48 263.24	13.32 34.71
LNS Oriscav-Lux2 (LU2)	2017	ns 123 s 25	33.5	48.0	68.42 184.85	14.44 28.47
LNS Oriscav-Lux2 (LU3)	2018	ns 12	33.7	58.3	57.93	11.69
ANSP ESTEBAN (FR1)	2014	ns 36 s 27	31.4	50	68.25 278.72	8.2 26.15
ANSP ESTEBAN (FR2)	2015	ns 138 s 64	32.5	39.9	85.94 304.32	10.35 29.29
ANSP ESTEBAN (FR3)	2016	ns 23 s 10	34.0	34.8	89.22 222.21	11.43 21.42

AAMA: N-acetyl-S-(carbamoylethyl)-l-cysteine), GAMA: N-acetyl-S-(1-carbamoyl-2-hydroxyethyl)-l-cysteine), non-smoker: n.s., smoker: s.

**Table 3:** Estimated slope (s) and statistical significance of a multiple regression for AAMA and GAMA in µg/L considering age and distribution of sexes as covariates and for populations including (top) or excluding (bottom) studies with values estimated from blood samples. Estimations from blood levels were not performed for GAMA (due to regional variance of estimation factors). ***: p<0.0001, **: p<0.001, *: p<0.05, n.s.: not significant.

	Counts (N)	Variable	AAMA vs. Sampling Year	GAMA vs. Sampling Year
including estimated values	5245	Sampling year	s: 2.09, ***	-
		Age	s: −0.49, **	-
		Sex	s: 0.21, n.s.	-
excluding estimated values	4202	Sampling year	s: 1.91, **	s: 0.12, n.s.
		Age	s: −0.45, *	s: −0.07, n.s.
		Sex	s: 0.12, n.s.	s: −0.06, n.s.

## Data Availability

Data visualizations will be made available at https://www.hbm4eu.eu/what-we-do/european-hbm-platform/eu-hbm-dashboard/ (last accessed on 26 July 2022).

## Abbreviations

The following abbreviations are used in this manuscript:

AAMA	N-acetyl-S-(carbamoylethyl)-l-cysteine)
GAMA	N-acetyl-S-(1-carbamoyl-2-hydroxyethyl)-l-cysteine)
HBM4EU	Human biomonitoring for European Union
CYP2E1	Cytochrome P450 2E1
